# Analyzing the characteristics of Otitis media with effusion following SARS-CoV-2 infection in China

**DOI:** 10.3389/fsurg.2025.1515724

**Published:** 2025-05-12

**Authors:** Xinxin Li, Yanfang Liu, Minxing Tan, Xuanfu Zeng, Muhammad Asad Iqbal, Guochang Jiang

**Affiliations:** ^1^ Department of Otolaryngology, Affiliated People’s Hospital of Jiangsu University, Zhenjiang, Jiangsu, China; ^2^ Laboratory Center, Affiliated People’s Hospital of Jiangsu University, Zhenjiang, Jiangsu, China; ^3^ School of Medicine, Jiangsu University, Zhenjiang, Jiangsu, China

**Keywords:** otitis media with effusion (OME), COVID-19, SARS-CoV-2, conductive hearing loss, mixed hearing loss, middle ear effusion (MEE)

## Abstract

**Objective:**

This study investigates the characteristics of Otitis Media with Effusion (OME) secondary to the SARS-CoV-2 pandemic, and examines whether SARS-CoV-2 is present in middle ear effusions (MEE).

**Methods:**

We analyzed patients diagnosed with SARS-CoV-2 who presented with ear fullness between December 15, 2022, and January 20, 2023. After obtaining a detailed medical history and conducting audiometric assessments, we confirmed OME and performed tympanocentesis to test for SARS-CoV-2 in the MEE following informed consent. Post-procedure, patients received nasal decongestants and oral/nasal corticosteroids. Follow-up consultations, tympanic membrane examinations, and audiometric evaluations were conducted 2–4 weeks later, with a final assessment at three months.

**Results:**

Our clinic recorded 311 OME cases during the study period, accounting for 9.5% of all patients—a significant increase from 2.2% the previous year and 2.5% the following year. The peak incidence occurred one week post-infection. Among the 311 patients, 52 underwent tympanocentesis (33 males, 19 females). 20 patients had bilateral onset, while 32 had unilateral onset. 31 patients were cured after a single tympanocentesis, whereas 21 required two or more procedures. 17 patients tested positive for SARS-CoV-2 in the MEE, but only one simultaneously tested positive in nasal secretions. At the three-month follow-up, 59.6% of patients were cured, 30.8% showed improvement without full recovery, and 9.6% had no improvement. Factors such as poor mastoid pneumatization, nasopharyngeal obstruction, and comorbidities (hypertension, diabetes) affected treatment efficacy. Among the 52 patients, 37 had conductive hearing loss (CHL), and 15 had mixed hearing loss (MHL).

**Conclusions:**

SARS-CoV-2 contributes to OME, primarily affecting one ear. The virus persists longer in MEE than in the upper respiratory tract, suggesting slower viral clearance in the middle ear compared to the nasopharynx. Conductive hearing loss (CHL) is the most common type post-infection, but mixed hearing loss (MHL) can also occur, particularly in older patients, with less favorable outcomes compared to CHL.

## Introduction

Otitis media with effusion (OME), characterized by sterile fluid accumulation in the middle ear, is a well-documented sequela of upper respiratory tract infections (URTIs). Its pathogenesis is classically attributed to Eustachian tube dysfunction (ETD), often secondary to adenoid hypertrophy in children or mucosal inflammation or nasopharyngeal obstruction in adults (1, 2). Pediatric populations are more susceptible due to their anatomically shorter, more horizontal Eustachian tubes and frequent viral exposures (3). In adults, OME is relatively uncommon follows sporadic URTIs, with most cases resolving spontaneously. However, the emergence of SARS-CoV-2 has reshaped this paradigm, introducing novel etiopathogenic mechanisms and epidemiological trends.

The COVID-19 pandemic, initially marked by severe lower respiratory involvement (e.g., viral pneumonia), evolved with subsequent variants (e.g., Omicron) to predominantly cause upper respiratory symptoms—nasal congestion, pharyngitis, and ETD (4–6). This shift became particularly evident after China's December 2022 transition from its “Zero-COVID” policy, which led to widespread Omicron infections. Clinicians observed an unprecedented surge in post-COVID-19 OME cases, with adults reporting acute-onset aural fullness and conductive hearing loss, often persisting weeks after typical symptoms resolved (7–10). Recent investigations have demonstrated that SARS-CoV-2 was detected in 12% of MEE samples (median detection time: 21 days post-infection), providing compelling evidence for direct viral pathogenesis (11). Although comprehensive treatment regimens achieved an 83.6% success rate, the observed 8.2% recurrence rate highlights OME as a clinically significant post-COVID-19 complication (12). This emerging association between SARS-CoV-2 and OME presents both diagnostic challenges and opportunities for improved management strategies.

Accumulating evidence suggests SARS-CoV-2 may directly invade the middle ear mucosa. The virus's affinity for angiotensin-converting enzyme 2 (ACE2) receptors, abundantly expressed in the eustachian tube and middle ear epithelium, provides a plausible mechanism for viral entry and localized inflammation (5).

This study aims to systematically investigate the clinical and pathological characteristics of OME in patients with recent SARS-CoV-2 infection. Specifically, we will evaluate therapeutic outcomes and investigate if there is presence of SARS-CoV-2 in MEE, thereby enhancing our understanding of the link between post- SARS-CoV-2 infections and OME. We also focus on the type of hearing loss and the recovery rate respectively.

## Materials and methods

This retrospective observational study analyzed patients presenting with unilateral or bilateral ear fullness following SARS-CoV-2 infection at Jiangsu University Affiliated People's Hospital otolaryngology clinics between December 15, 2022 and January 20, 2023 (37-day period), with ethics approval (SQK-20240156-W). We examined SARS-CoV-2 diagnosis timing/method (antigen/nucleic acid test), OME onset timing, laterality (unilateral/bilateral), duration, accompanying tinnitus, and associated symptoms including fever, nasal congestion/rhinorrhea, cough (with/without sputum), and olfactory/gustatory dysfunction. Evaluations included pure-tone audiometry, tympanometry, endoscopy, optional CT scans, and prognostic outcomes.etc.

### Inclusion criteria

Patients were enrolled if they met (1) Primary complaint: Persistent ear tightness post–SARS-CoV-2 infection. (2) Audiometric evidence: Conductive or mixed hearing loss with “B"/"C” tympanogram. (3) Otoscopic findings: Tympanic membrane inversion or MEE. (4) Consent for procedures: Willingness to undergo tympanocentesis with SARS-CoV-2 testing of MEE. (5) Follow-up compliance: Completion of scheduled follow-ups.

### Exclusion criteria

Patients were excluded if they had (1) History of nasopharyngeal cancer or untreated nasal polyps; (2) Recurrent OME; (3) Refusal of SARS-CoV-2 nucleic acid testing in MEE; (4) Loss of follow-up.

### Treatment

Patients who enrolled underwent the following stepwise therapeutic protocol: (1) Tympanocentesis: All patients received tympanocentesis as the primary intervention to evacuate middle ear effusion. (2) Adjunctive Nasal Decongestant Therapy: Intranasal oxymetazoline 0.05% (or equivalent) was prescribed post-procedure. Dosage: 1–2 drops into the affected nostril(s), three times daily. Duration: Limited to 7 consecutive days. (3) Corticosteroid Regimen: First-line: Oral prednisone (0.5 mg/kg/day) was initiated for 5–7 days unless contraindicated (e.g., diabetes, uncontrolled hypertension). Alternative: For patients with contraindications to systemic corticosteroids, nasal spray corticosteriod (2 sprays per nostril twice daily) was substituted for 7–14 days.

Follow-up evaluations occurred at 2, 4, and 12 weeks post-treatment to assess therapeutic response and complications.

### Efficacy evaluation

Patients underwent clinical re-evaluation within 2–4 weeks post-treatment. Those not achieving cure received additional tympanocentesis as well as nasal spray coriticosteriod for more than 2 weeks, and outpatient follow-up at 3 months. Treatment efficacy was assessed based on symptomatic improvement and audiometric recovery. Following sudden deafness prognostic criteria, outcomes were categorized into three grades: (1) Cured—hearing restoration to normal levels (pure-tone average ≤25 dB HL) with Type A tympanogram; (2) Effective—hearing improvement >15 dB HL without reaching normal levels, with Type B/C tympanogram; and (3) ineffective—hearing improvement <15 dB HL, with persistent Type B/C tympanogram.

### Statistical analysis

Statistical analysis was performed using GraphPad 7, t-test and two-way ANOVA were used, *P* < 0.05 was statistically significant, and the continuous data were expressed as mean ± standard deviation.

## Results

### The impact of SARS-CoV-2 on the incidence and characteristics of OME

**Figure 1:**
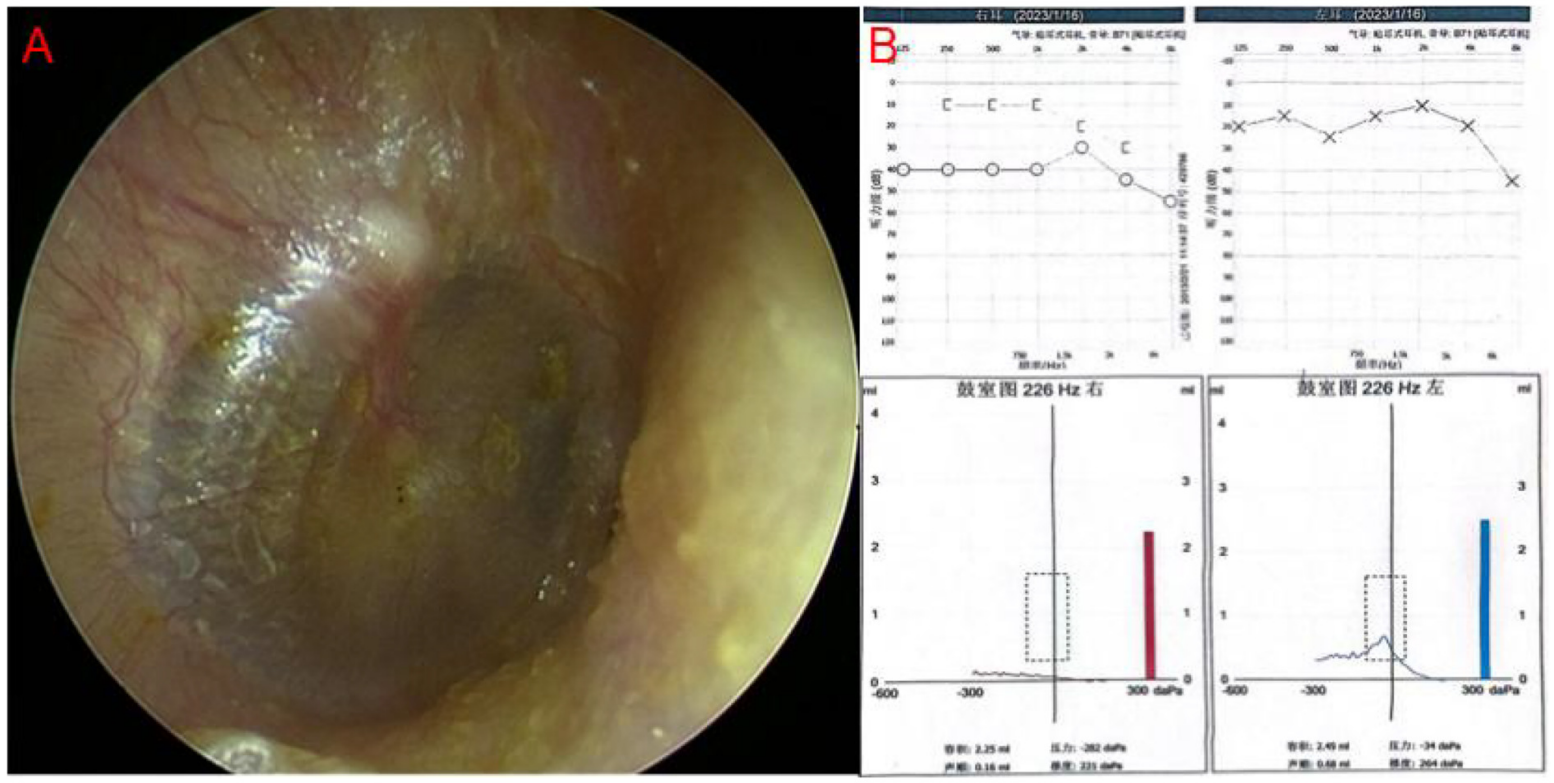
**(A)** Endoscopic image of typical OME, showing yellowish fluid in the tympanic chamber. **(B)** Pure tone audiometry and tympanometry in typical OME. There is a gap between air conduction and bone conduction curve in the right ear, tympanometry shows type B curve in the right ear.

**Figure 2:**
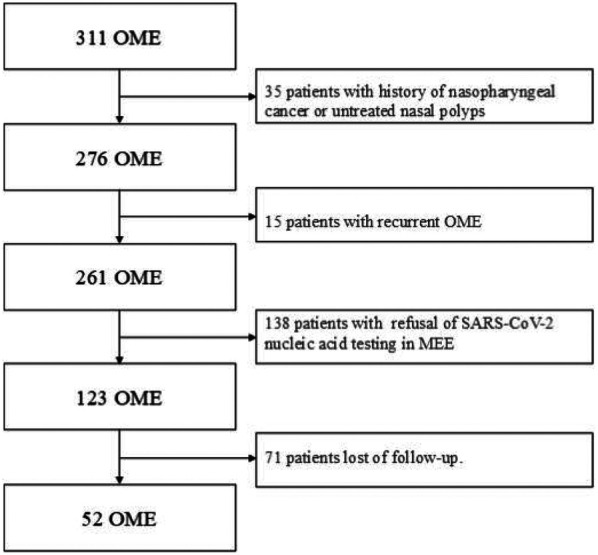
Flow chart with patients in this study.

**Figure 3:**
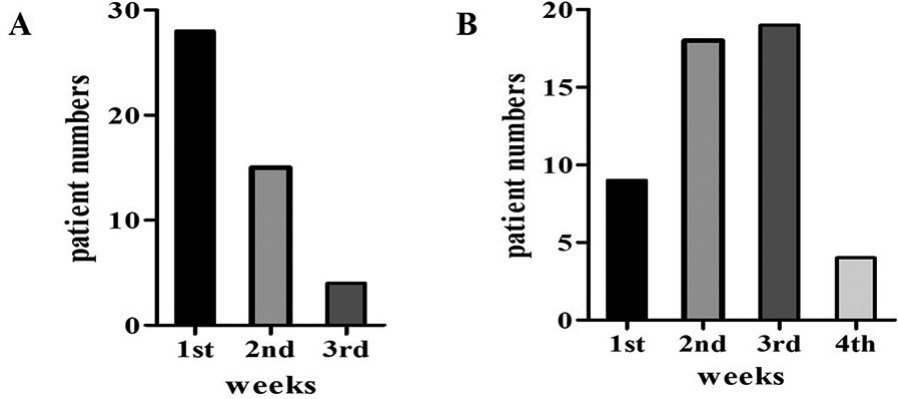
**(A)** The onset of ear fullness post-infection. **(B)** Time for medical visits post-infection.

311 patients with otoscope, pure tone audiometry, and tympanometry were confirmed as OME secondary to SARS-CoV-2 infection (Figure 1). This accounts for 9.5% of all diseases in the Otolaryngology clinic during the same period, the incidence rate has increased considerably in comparison to the 2.2% prevalence in the same period of 2021, and 2.5% prevalence in the same period of 2023. Among the 311 patients, 138 declined SARS-CoV-2 nucleic acid testing in MEE, 71 lost of follow-up, 35 had a history of nasopharyngeal cancer or untreated nasal polyps, and 15 were excluded due to recurrent OME, 52 patients were enrolled in the study (Figure 2). There were 33 males, and 19 females, who are from 29 to 86 years old, average 54.9 ± 14.2 years old. 20 individuals got binaural involved, which accounted for 38.5% of the total, while 32 individuals got monaural involved, which accounted for 61.5%. The peak of the onset of OME occurs within one week following a large-scale SARS-CoV-2 infection in December 2022 (Figure 3A). The peak of seeking medical treatment occurred 2–3 weeks' post-infection (Figure 3B). 21 patients experienced at least twice tympanocentesis which accounted for 40.4% (Table 1).

**Table 1:** The characteristics of the OME secondary to SARS-CoV-2 infection.

Category	Numbers/percentage (N)
Age	54.9 ± 14.2
Gender	
Male	33 (63.5%)
Female	19 (36.5%)
Affected side	
Bilateral	20 (38.5%)
Unilateral	32 (61.5%)
Tympanocentesis	
Once	31 (59.6%)
Twice or more	21 (40.4%)
COVID-19-19 in the Middle Ear	
Positive	17 (32.7%)
Negative	35 (67.3%)

### SARS-CoV-2 can be detected in MEE

**Figure 4:**
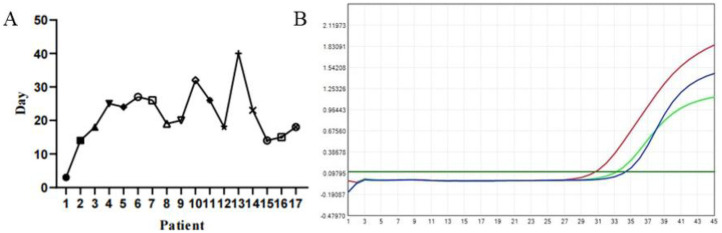
**(A)** Duration of ear fullness in patients with COVID-19 positive in MEE, the longest time last 40 days. **(B)** Positive for SARS-CoV-2 nucleic acid in MEE by polymerase chain reaction (PCR) testing.

Among 52 patients undergoing tympanocentesis with concurrent SARS-CoV-2 testing of both middle ear effusions and nasal secretions, 17 (32.7%) showed viral presence in middle ear effusions (Figure 4B). Notably, only one of these 17 patients simultaneously tested positive in nasal secretions. Persistent viral detection was observed in one case with ongoing ear fullness lasting 40 days (Figure 4A). These findings demonstrate significantly prolonged viral persistence in the middle ear compared to the nasal cavity, suggesting slower viral clearance from the middle ear space.

### Comparative analysis of hearing threshold changes in conductive and mixed hearing loss patients pre and post treatment

Analysis of pure tone audiometry and tympanometry results revealed 37 patients (71.2%) with conductive hearing loss (CHL) and 15 patients (28.8%) with mixed hearing loss (MHL) (Table 2). The MHL group showed higher prevalence among elderly patients (mean age: 67.80 ± 10.29 years). Post-treatment analysis demonstrated significant improvements in both air conduction (AC) and bone conduction (BC) thresholds: CHL patients showed AC improvement from 42.38 ± 12.16 dB HL to 22.86 ± 9.16 dB HL (*P* < 0.0001) and BC improvement from 15.76 ± 3.91 dB HL to 13.35 ± 2.83 dB HL (*P* = 0.0034), while MHL patients exhibited AC improvement from 76.40 ± 21.18 dB HL to 57.13 ± 23.88 dB HL (*P* = 0.0268) but non-significant BC change (46.00 ± 15.38 dB HL to 38.00 ± 14.84 dB HL, *P* = 0.1583). Intergroup comparisons showed significant differences in both post-treatment AC (*P* < 0.0001) and BC thresholds (*P* < 0.0001) (Table 3), confirming greater treatment efficacy for CHL patients, particularly in air conduction improvement, with MHL patients showing less pronounced BC threshold benefits.

**Table 2:** Rate of effective between the two groups.

Type of HL	Therapeutic effect			
	Cured	Effective	Ineffective	Rate of effective
CHL (37)	31	6	0	100%
MHL (15)	0	10	5	66.70%

**Table 3:** The hearing level in conductive hearing loss (CHL) and mixed hearing loss (MHL) pre-treatment and post-treatment, which include air conduction (AC) and bone conduction (BC).

Type of HL	Pure-tone HL					
	AC		*p*	BC		*p*
	pre-treatment (dB)	post-treatment (dB)		pre-treatment (dB)	post-treatment (dB)	
CHL	42.38 ± 12.16	22.86 ± 9.16	<0.0001	15.76 ± 3.91	13.35 ± 2.83	0.0034
MHL	76.40 ± 21.18	57.13 ± 23.88	0.0268	46.00 ± 15.38	38.00 ± 14.84	0.1583

### Impact of nasopharyngeal lesion and other factors on hearing outcomes in patients with OME post- SARS-CoV-2 treatment

At the follow-up visit after 3 months, 31 patients were cured, accounting for 59.6%; 5 ineffectives, accounting for 9.6%; There were 16 partially improved, accounting for 30.8%. Post treatment, 3 of the 5 ineffective patients were positive for the SARS-CoV-2 in MEE. Among the patients, one exhibited poor mastoid pneumatization, one had uncontrolled hypertension, and one was diagnosed with diabetes. 10 patients in the MHL group who improved but did not recover (effective), the hearing test results showed that in addition to conductive hearing loss, sensorineural hearing loss was also present, and there were underlying diseases such as poor mastoid vaporization, dysfunction of the Eustachian tube, hypertension, diabetes, etc.

## Discussion

Our study demonstrates a clear temporal association between China's Omicron-dominant COVID-19 phase and a significant increase in OME incidence, with cases accounting for 9.5% of otolaryngology visits compared to baseline levels of 2.2%–2.5% This epidemiological shift, peaking 1-week post-infection with delayed clinical presentation (2–3 weeks), establishes OME as an important post-COVID-19 sequela. Notably, we identified prolonged viral persistence in middle ear effusions (32.7% PCR-positive) compared to concurrent nasopharyngeal samples (1.9%), suggesting the middle ear may serve as a viral reservoir even after upper respiratory tract clearance. The predominance of conductive hearing loss (71.2%) vs. mixed hearing loss (28.8%)—the latter carrying poorer prognosis particularly in elderly and comorbid patients—provides the first clinical stratification of post-COVID-19 auditory complications. These findings have immediate implications for otologic practice: The results highlight the need for: (1) routine middle ear monitoring in post-COVID-19 patients with ear symptoms, (2) prompt tympanocentesis for high-risk groups (elderly, diabetics, or those with mastoid abnormalities), and (3) customized follow-up based on hearing loss type. At a public health level, these data argue for including OME in post-COVID-19 syndrome monitoring frameworks and anticipatory resource allocation during future infection waves. The 9.6% treatment-refractory rate underscores the need for research into therapies targeting viral persistence mechanisms in the middle ear.

Previous studies have detected SARS-CoV-2 in various bodily fluids (13–15). Our finding of viral RNA in MEE samples suggests the eustachian tube may serve as a conduit for viral spread from the nasopharynx, consistent with Wu et al.'s report of higher nasopharyngeal viral loads (16). The virus may migrate retrogradely through the eustachian tube, which contains abundant ACE2-rich ciliated cells (17–19).

In one patient from our study, SARS-CoV-2 was detectable in the MEE 40 days post-infection, even though the nasopharyngeal swab had already tested negative. This suggests that the virus persists longer in the middle ear than in the upper respiratory tract, complicating treatment outcomes, particularly in patients history of similar episodes (20). However, despite the detection of SARS-CoV-2 in MEE, the Ct value of 37.30 indicates that its viability and pathogenicity had significantly decreased.

Clinically, patients typically developed fever and fatigue initially, followed by throat pain/cough within 1–2 days, and ear fullness approximately one week later—a progression consistent with eustachian tube anatomy. While the precise OME pathogenesis remains unclear, SARS-CoV-2's presence in the middle ear and ACE2 receptor distribution support potential direct infection or tubal dysfunction mechanisms (19, 21).

Of the 52 patients who underwent tympanocentesis, 59.6% required only one procedure, while 40.4% underwent multiple procedures. Additionally, 38.5% of the cases were bilateral. After three months of follow-up, 59.6% of patients were cured, 9.6% showed no improvement, and 30.8% were partially improved. Comorbidities, such as hypertension and poor mastoid pneumatization, were associated with poorer treatment outcomes, aligning with previous studies that have noted the influence of underlying health conditions on otologic complications (22, 23).

In terms of hearing outcomes, conductive hearing loss was the most common, although a few cases of mixed hearing loss were also observed. Our study showed that patients with mixed hearing loss generally had poorer prognoses compared to those with conductive hearing loss, highlighting the need for targeted audiological assessments. How does the COVID-19 impact on the inner ear? One hypothesis is that the affection of COVID-19 may lead to a hypercoagulable state of blood in the inner ear vessels, which is eventually caused cochlear ischemia, then hearing loss or even deafness may occur (24). Most cases of mixed hearing loss occurred in elderly patients (average age 67.8 ± 10.29), OME of whom may have had pre-existing mild sensorineural hearing loss. However, previous studies have speculated that SARS-CoV-2 infection could be a sole cause of sudden sensorineural hearing loss, raising the possibility that COVID-19 may contribute to nerve-related hearing loss, which presents as mixed hearing loss when combined with MEE (25, 26).

Interestingly, while viral infections are a common cause of OME, particularly in children due to the shorter and straighter structure of their eustachian tubes, we observed fewer cases in children during the pandemic, with a marked increase among adults (27). This could be attributed to the milder systemic symptoms of COVID-19 in children compared to adults.

This study has several limitations that warrant consideration. First, the retrospective design may introduce potential biases in data interpretation. Second, as a single-center study with a limited sample size, the generalizability of our findings requires further validation. Additionally, the 3-month follow-up period may be insufficient to evaluate long-term outcomes, and more comprehensive audiological assessments (e.g., repeat pure-tone audiometry and tympanometry) would strengthen the reliability of the results. Future multicenter prospective studies with extended follow-up durations and standardized audiological evaluations are needed to confirm our findings and further explore the long-term efficacy and safety of the intervention.

## Conclusion

In summary, our study highlights the otologic manifestations of COVID-19 and suggests that middle and/or inner ear involvement may be an underrecognized complication. This finding reinforces the need for surveillance of post-COVID otologic complications, particularly in high-risk populations. Long-term follow-up studies should assess whether SARS-CoV-2-related OME increases the risk of chronic middle ear disease or hearing impairment. Further investigations should explore: (1) the precise mechanisms of viral entry into the middle ear, (2) the pathophysiological basis for sensorineural hearing loss (SNHL) development, and (3) evidence-based optimization of therapeutic strategies.

## Data Availability

The raw data supporting the conclusions of this article will be made available by the authors, without undue reservation.
